# Using Online Photovoice to Explore Food Decisions of Families on Low Income: Lessons Learnt During the COVID-19 Pandemic

**DOI:** 10.1177/10497323231208829

**Published:** 2023-11-07

**Authors:** Eleni Spyreli, Elena Vaughan, Michelle C. McKinley, Jayne V. Woodside, Marita Hennessy, Colette Kelly

**Affiliations:** 1 Centre for Public Health, 1596 Queen’s University Belfast, Belfast, UK; 2 Institute for Global Food Security, 1596 Queen’s University Belfast, Belfast, UK; 3 Health Promotion Research Centre, School of Health Sciences, 8799 University of Galway, Galway, Ireland; 4 College of Medicine and Health, 8795 University College Cork, Cork, Ireland

**Keywords:** photovoice, online interviews, families on low income, food decisions

## Abstract

The method of photovoice has been previously used to effectively engage with socioeconomically disadvantaged groups and explore their eating behaviours. In this methodological article, we draw on our experiences from using photovoice through online interviews with families on low income about their food decisions. A purposive recruitment approach targeted parents of children 2–17 years old who lived on a tight budget across the island of Ireland. Participants provided demographic information and were invited to take photographs of food-related decisions and activities for 1 week during the COVID-19 lockdown. The photographs were then discussed through an online communication platform to generate qualitative data. A total of 28 parents participated in the photo-elicited interviews and shared a total of 642 photographs of factors that influenced their food decisions. Following the interviews, the researchers documented their reflections which focused on (1) participants’ engagement with the online photo-elicitation and (2) practical aspects around participant consent and data safety. The participants in our study engaged well with the online photovoice method and shared a variety of photos which provided ample material to facilitate the conversations around their food environment and its impact on their food decisions. Our experiences can provide novel insights into using photovoice in a virtual environment and useful considerations around ethics and data collection for researchers who work with socioeconomically disadvantaged groups. Photo-elicited interviews offer an engaging and flexible data collection technique that can highlight issues informing future priorities of healthcare policy.

## Introduction

Low-income households on the island of Ireland (i.e. Northern Ireland and Republic of Ireland) consume suboptimal diets that are characterised by a high intake of total fat and saturated fat and low consumption of fruit and vegetables (Bates et al., 2014; Bates et al., 2019; Healthy Ireland, 2016). During the COVID-19 pandemic, the dietary habits of these households may have deteriorated due to a reduced availability of foods (OECD, 2020), increased food prices (Office for National Statistics, 2021) and the need of individuals with underlying health issues to stay indoors to protect themselves from the spread of the virus. Additionally during this time, food charity networks saw a rise in the use of food banks with low-income families relying heavily on them (The Trussel Trust, 2021; Food Cloud, 2020). COVID-19 represented an unprecedented shock to the macro-level system which disproportionately affected those experiencing social and economic precarity. Such challenges are indicative of the structural and environmental influences on food-related practices and behaviours, particularly on vulnerable groups, and warrant exploration in terms of effects on dietary behaviours and their potential health impacts.

Individuals at socioeconomic disadvantage (i.e. experiencing inequalities related to education, income and access to healthcare) and those at risk of disadvantage (e.g. lone parents, migrants and people with disabilities) are often excluded from participating in health promotion research (Lowrie & Tyrrell-Smith, 2017). Barlow and colleagues highlight that many barriers to the participation in research of families experiencing deprivation include its lack of relevance or perceived benefit to their lives (Barlow et al., 2005). Lack of trust of researchers has also been quoted as a possible reason for refusal to engage in research (Coe et al., 2008). For culturally and linguistically diverse groups, research may be seen as irrelevant or inappropriate to meet their needs (Cortis et al., 2009). Practical barriers, such as lack of time and transport, can be added to the circumstances experienced by low-income families that lead to their exclusion from research (Boag-Munroe & Evangelou, 2012). Consequently, their voices are rarely heard and, hence, not reflected within public health and nutrition policy, which can further exacerbate health inequalities (Richards, 1998; Marmot, 2015).

Research in most disciplines requiring face-to-face data collection was significantly impacted by the COVID-19 restrictions (Johnson et al., 2021; Lupton, 2020), and researchers needed to consider alternative approaches to in-person data collection. Although the use of remote technologies for conducting research preceded the COVID-19 pandemic (Lo Iacono et al., 2016; Weller, 2017; Seitz, 2016; Woodyatt et al., 2016), it came to the forefront during the COVID era as often the only way for researchers to mitigate the challenges of lockdown. A growing literature base has arisen during the pandemic, documenting the adaptation to online technologies which have provided researchers with novel and practical tools to engage participants in research (Dodds & Hess, 2020; Rania et al., 2021; Bania & Dubey, 2020; Archibald et al., 2019). Conducting research online allows researchers to engage with participants from geographical areas difficult to access (Rania et al., 2021; Archibald et al., 2019). It also enables interviewees to participate in research from the comfort of their home and to share sensitive information whilst maintaining a certain level of anonymity, when compared with face-to-face interactions (Rania et al., 2021; Bania & Dubey, 2020). On the other hand, online interviews lack elements of non-verbal communication that facilitate rapport and may exclude individuals who are not familiarised with online technologies (Dodds & Hess, 2020; Rania et al., 2021; Bania & Dubey, 2020). With these challenges in mind, existing literature indicates that research methodologies can be effectively adapted to a virtual environment and online platforms can offer useful tools to carry out research in circumstances similar to the COVID-19 lockdown.

The switch to web-based platforms due to the requirements of COVID lockdown also provided researchers with a window of opportunity to test novel interviewing techniques that encourage participant interaction. Photovoice is a participatory visual research methodology, introduced by Wang and Burris (1997) and applied to public health and health promotion. Participants engaging in photovoice are invited to photograph scenes, people, or objects pertinent to them and the research topic and, subsequently, discuss the photos with other participants or the researcher(s). Photovoice is an effective way to engage with groups and communities that are marginalised (due to ethnicity, geographical location and socioeconomic deprivation) and enables them to capture issues of concern to them and communicate their perspectives and experiences, which otherwise would have been overlooked by research and policy makers (Wang & Burris, 1997). Photovoice is a powerful tool used in explorations in various disciplines, for example, to help understand complex psychosocial issues (Cordisco, 2022; Kim et al., 2015; Niepage et al., 2018) or to highlight issues of health inequities (Kovacic et al., 2014; Augsberger et al., 2022; Mejia et al., 2013; Haque & Eng, 2011). It is also well suited to the exploration of eating behaviours and food security, and it has been previously used to provide insights into participants’ perspectives of daily dietary decisions and concerns (Brown & Holloway, 2008; Martin et al., 2010; Thomas & Irwin, 2013; Chilton et al., 2009; Garcia et al., 2010; Valera et al., 2009; Share & Hennessy, 2017).

## Research Aim

This methodological article describes our experiences gained from, and reflections on, utilising photovoice methodology through online interviews with low-income families on the island of Ireland about their food-related decisions. We hope that lessons learnt through the conduct of this study will provide useful considerations for researchers who wish to maximise the use of remote participatory methodologies for data collection in public health and other research disciplines.

## Research Overview

This work is part of a broader study that explored (1) how low-income families navigate their local food environment; (2) which environmental factors contribute to their food choices; and (3) what is the impact of COVID-19 lockdown on these choices. The protocol and some of the findings of the initial study have been previously described (Safefood, 2022).

### Patient and Public Involvement

Prior to the commencing of the study, a panel of parents living in the Republic of Ireland provided input into the research questions, tools, recruitment and dissemination material and approaches. They were selected through the same channels utilised for recruitment in the main study to ensure that the families advising on the research have similar experiences and living conditions to those participating. This collaborative approach was guided by the principles of public involvement in research (Palm et al., 2015).

### Ethical Considerations

The study was originally approved by the Research Ethics Committees of both institutions involved in this work. The initial protocol outlined the use of face-to-face interviews, but the COVID-19 pandemic and public health restrictions meant this was impossible. Thus, an amendment to the ethics application was made to pivot to online recruitment and data collection and to add a question about the impact of COVID-19. Considerations including choice of a secure communication software, data storage and privacy were documented in the amendment application; these are described below.

### Sampling and Recruitment

An online purposive snowball sampling approach was used. Researchers drew on existing community contacts who worked with parents and could share the study information through their mailing lists/networks. Social media channels (Twitter, Facebook, LinkedIn, Instagram) were also utilised to further disseminate the call for participation. Interested individuals were invited to contact the research team to express interest in the study.

Eligible participants were parents or guardians of children 2–17 years old who lived in rural and urban areas on the island of Ireland (i.e. Republic of Ireland (RoI) and Northern Ireland (NI)). Both fathers and mothers were invited to participate; the same recruitment channels and materials were used to target both genders. The study aimed to capture the perspectives of families on a low-income and, therefore, from the parents who expressed interest, only those who self-identified as ‘living on a tight budget’ were recruited. The purposive sampling strategy ensured that a variety of perspectives were included, such as those of single-parent and two-parent households and parents with children in the age group of 2–12 and 12–17 years.

A participant information sheet was sent electronically to all eligible individuals, which outlined the study aim and what participation entailed. It also introduced the principles of photovoice (see Supplementary File 2). After sending the information, researchers waited for 48 hours before following up with eligible individuals to see if they were interested in taking part.

### Introducing Photovoice to Participants

After identifying eligible participants, the research team obtained informed consent. Parents were asked for their permission to participate in the research and to use their photographs in future publications and presentations on the research findings. They were also informed about confidentiality and their right to withdraw at any point and to ask for their photographs to be deleted, up to the point of anonymisation, without providing a reason for doing so. An electronic copy of the consent form was sent to them via email which they were asked to sign and return again via email. Following receipt of consent, a first short call was arranged based on participants’ availability.

In this introductory phone call, researchers provided a summary of the study aim and what participation would involve and proceeded to capture participants’ demographic information. The methodology of photovoice was also described in detail, and participants were asked to use their personal digital or phone cameras to take photographs of food-related decisions and activities for 1 week during the COVID-19 lockdown. As general guidance, researchers advised participants to take 1–2 photos per day, but simultaneously encouraged them to capture anything they thought relevant, along with general advice to avoid taking identifiable images of people to maintain anonymity. A few written examples were given in the participant information sheet of what the photographs could illustrate, such as offers on food, marketing material or receipts (see more examples in Supplementary File 2). These examples were kept to a minimum in case they restricted participants’ ideas.

Researchers also offered to send daily or less frequent text messages to parents to remind them to take photographs. Finally, participants were advised to share their photos with the researchers once they had completed the task. The two institutions involved in the study differed in their procedures in relation to photo sharing (through email or WhatsApp) due to differing data processing and security requirements. Participants were encouraged to ask questions and seek clarifications at any point leading up to (and during) the photovoice interview.

### Online Photo-Elicited Interviews

An interview was arranged based on participants’ availability approximately a week after the first call. When arranging the interview, researchers also took into consideration the times that participants could be alone at home or in a separate room away from the other family members in order to maintain the privacy of the discussions. One day before the scheduled interview, researchers sent out a text message to confirm participants’ availability for the interview and to remind them to share their photos, if they hadn’t done so already. Participants’ photographs were compiled and organised in a folder or in a PowerPoint file in advance of the photo-elicitation interview to facilitate online photo sharing (by the host/researcher) and minimise disruption during the course of the conversation.

Interviews were conducted online through the communication platform *Microsoft Teams* (Microsoft Corporation, Redmond, WA, US), which was chosen as per suggestion of the University Data Governance team for security reasons. Logging into an *MS Teams* call is possible through following a unique link generated for each meeting, for each participant in this case, since interviews were one to one. Additionally, by enabling the ‘lobby’ function (i.e. a virtual waiting area for guests), the meeting host can screen anyone who clicks on the meeting link before allowing them to join the meeting; this aspect prevents anyone else entering the meeting. *MS Teams* also gives users the option to participate in a call through their phone, tablet or computer and to share only audio or audio with image. In the case that participants’ Wi-Fi connection dropped, or the software failed, a regular phone call was considered an alternative plan.

Researchers started the interview by introducing themselves, thanking parents for taking part in the study and reminding them of the purpose of the interview. Their camera was activated for this initial stage but deactivated for the remainder of the interview, where the focus was on the photographs. Parents were encouraged to have their cameras on, only if they wished to. Their photographs were then discussed one by one as they were presented by the researchers in real time with the share screen function. In cases where participants sent slightly different versions of the same photo, the photo was discussed only once. Conversations were facilitated by a brief topic guide that helped elicit a meaningful and comprehensive story from every photograph (see Supplementary File 1). Participants were also encouraged to add their own insights. The methods were previously piloted with three participants from the RoI to ensure that the method was feasible and appropriate for the target sample.

All interviews were moderated by researchers with previous experience in qualitative interviews (*researchers’ initials omitted for double-anonymised peer review*) and were audio-recorded on *Teams*. Discussions were transcribed verbatim in the following process: recordings were used to generate transcripts through the relevant function offered by the *Teams* software, with the consent of participants; these transcripts were then checked against recordings by the researchers to produce a more accurate verbatim record (i.e. correct any wording mistakes and add punctuation). All identifiable information such as names was removed from the transcripts; a pseudonym was allocated to each participant.

### Distress Protocol

It was deemed possible that the topics discussed during these interviews may be distressing for some parents (e.g. weekly budget for food), and therefore, they were reminded that they did not have to answer a question that made them feel uncomfortable. Additionally, whenever entering a topic that may be sensitive or provocative, the researchers prefaced the question with asking for permission, that is, ‘*Do you mind if I ask you a question about x or y*?’. Participants were reminded that they could leave the interview at any time, or have a break, and were not obliged to speak about anything they did not wish to. Additionally, a distressed participant protocol was developed to help researchers deal with possible situations where participants became upset.

## Results

The qualitative researchers documented their reflections, as these arose from notes when drafting the amended submission to the Ethics Committee; discussions within the wider research team; and fieldworkers’ research journals. Their notes were reviewed and considered for this article. Together with the other members of the research team, reflections on the process were discussed and perspectives were agreed. These perspectives are presented in two main sections in relation to (1) participants’ engagement with the online photovoice method, as perceived by researchers, and (2) practical aspects around participant consent and data safety.

### Participants’ Engagement With Online Photovoice

A total of 28 parents were interviewed between October 2020 and February 2021; 12 lived in NI and 16 in the RoI. Although participation was open to both males and females, there were many more mothers than fathers in the overall sample (*n* = 26), with only two fathers taking part. The majority had been born on the island of Ireland (64%) and were eligible for state benefits (79%). All study participants used their mobile phones for the purpose of the online photovoice interviews after downloading the *MS Teams* application. All of them reported being familiar with videoconferencing software through home-schooling or work, and some of them reported using the *Teams* software in the past.

**Figure 1.**
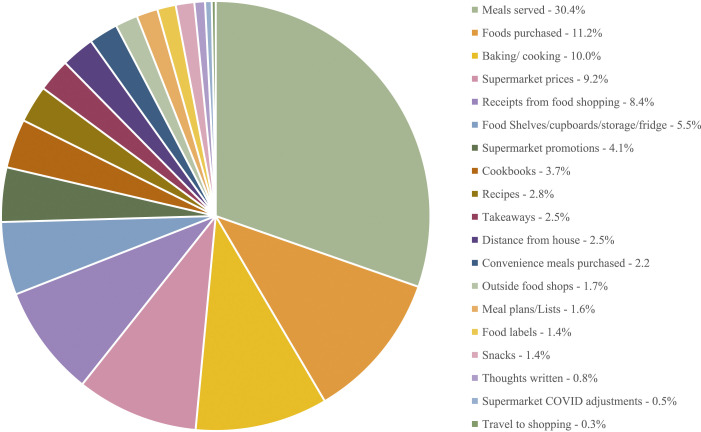
Categories of photos sent by participants.

Interviews lasted between 35 and 60 minutes. Participants in both jurisdictions sent a total of 642 photographs in advance of the interview. Each participant sent 23 photos on average, ranging from 3 to 135 per participant. The majority of participants’ photos portrayed family meals served at home (*N* = 195, 30% of total number of photos); followed by packaged foods purchased in shops (*N* = 72, 11%); the cooking/baking process (*N* = 64, 10%); and supermarket prices (*N* = 59, 9%). A breakdown of the categories of photos discussed in the interviews can be found in Table 1 and Figure 1. In cases where participants sent a very large number of photographs, the researchers selected a few that were representative of each category. Examples of participants’ photos can be seen in Supplementary File 3.

**Table 1.** Number and Frequency of Different Types of Photos Sent by Participants (Organised From Most to Least Frequent).

Categories of photos	*N*	%
Meals served	195	30.4
Foods purchased	72	11.2
Baking/cooking	64	10.0
Supermarket prices	59	9.2
Receipts from food shopping	54	8.4
Food shelves/cupboards/storage/fridge	35	5.5
Supermarket promotions	26	4.1
Cookbooks	24	3.7
Recipes	18	2.8
Takeaways	16	2.5
Distance from house	16	2.5
Convenience meals purchased	14	2.2
Outside food shops	11	1.7
Meal plans/lists	10	1.6
Food labels	9	1.4
Snacks	9	1.4
Thoughts written	5	0.8
Supermarket COVID adjustments	3	0.5
Travel to shopping	2	0.3

The process of photovoice was well understood based on discussions with participants and our observations as researchers. Participants confirmed that the explanation of the method provided during the first call was clear and that they did not require any further clarification. During the first contact, participants were also asked whether they wished to receive daily or less frequent text messages reminding them to take photographs for the photovoice activity throughout the week prior to the interview. Nobody availed of it however, citing that they did not deem it necessary. All participants who attended the introductory call proceeded to complete the photo-elicitation interview and sent their photos one day prior to it.

All participants took pictures through their personal mobile phones and directly sent them to the researchers through email or WhatsApp. All participants had a good internet connection that allowed logging successfully onto the *MS Teams* call and completing the interview with no interruptions. Participants chose a date and time when they could be at home on their own or in a room away from the other members of their family, with the exception of a single father who had to leave the interview for 5 minutes to tend to his daughter. The majority of participants shared only audio with their own camera off. Some chose to have their cameras on but were moving during some parts of the interview or were not entirely within the camera frame.

Participants’ photographs provided sufficient prompts to spark discussion around their food-related decisions, and researchers did not have to rely too much on the topic guide. All participants were keen to discuss their photos, independent of the number of pictures initially sent to the researchers. For every photo discussed during the interview, participants gave information about the wider context within which a food decision was taken, such as reliance on benefits and healthy start vouchers, children’s fussy eating, hectic work schedule, immigration, presence or lack of social support for single parents and other aspects of their lives (Safefood, 2022).

### Practical Aspects Around Participant Consent and Data Safety

The consent form was sent to all participants as an MS Word document. Most of them initialled and signed the document on their phone and emailed it back to the researchers. Some also took pictures of their hand-written signature and sent it to the researcher along with the consent form. In a few instances, parents printed the consent form which they then completed by hand, scanned (or photographed) and emailed back in a jpeg or pdf format. Consent forms were kept separately from the data collected during the two interviews, as they held personal identifiers.

Participants were encouraged to send their pictures via email or WhatsApp to the researchers. Email proved cumbersome for some participants, especially those who sent a large number of photos, which exceeded in size the maximum capacity of an email attachment. Hence, many participants chose to send their pictures through *WhatsApp* by using the phone number (research-specific phone) they were given to contact the research team. Additionally, some of the participants chose to send their photos through *WhatsApp* due to convenience and familiarity with the software.

Photographs sent through *WhatsApp* and email were transferred to a folder in an online university-shared drive (*MS OneDrive*), which only members of the research team had access to. The researchers then deleted the photographs from the study phone and their inbox. All other data collected (i.e. demographic information, consent forms and transcripts) were password protected and stored in the online-shared drive. The audio recordings of the online interviews were deleted, once the transcripts were generated. As instructed, participants refrained from capturing people in their pictures.

## Discussion

In this study, we explored the use of photovoice as a method of generating qualitative data for the purpose of public health nutrition research and as a method of engaging people who self-identify as living on a tight budget. Furthermore, we looked at the practical and ethical considerations related to photo-elicited interviews hosted in a virtual environment. Participants in this study showed good understanding of online photovoice and engaged well with the method, independently of the number of photos shared. The majority shared a variety of photos of their food environment which provided ample material to facilitate the conversations around participants’ food environment and its impact on their food decisions. The following paragraphs focus on our reflections on conducting a photovoice project that may provide important considerations to anyone who wishes to employ participatory data collection techniques including when, though not limited to, utilising web-conferencing platforms.

### Unlocking the Potential of Photovoice to Generate Data on Issues of Importance

Photovoice allowed for collecting rich qualitative data. Participants generally went on to discuss their photographs without any prompts, offering an unobstructed narration of everyday experiences related to food that quantitative methods or a rigid qualitative data collection protocol (e.g. structured topic guide) would not allow for. Alongside their food-related experiences, participants often openly talked about cultural and social constructs and the circumstances of their everyday lives that add valuable contextual information on how low-income parents made their food-related decisions. Several examples in the photovoice literature support its ability to generate rich descriptive information that researchers can use to promote health (Catalani & Minkler, 2010) and to reduce diet-related disparities (Valera et al., 2009; Colon-Ramos et al., 2018; Lardeau et al., 2011; Neill et al., 2011).

In this exploration, participants’ unobstructed narration was possibly facilitated by the fact that participants were not given rigid guidance on photography or an exhaustive list of images to capture. As Harrison points out (2002), providing participants with photography training or insisting on a certain kind of pictures might alter their practices of representation and reflections on the topic of inquiry.

Additionally, in this study, there was a very large variation in the number of photographs the participants sent. Interestingly, a couple of participants took more than 100 photos which made it impossible for the researcher to cover them all during the photo-elicitation, so a certain amount of selection and curation beforehand was necessary. Even though this curation was led by the researcher who facilitated the interview, it could also be done by the participants. This would ensure that the participants prioritise the issues discussed further empowering them in line with the photovoice ethos. In previous studies that have utilised photovoice, researchers have asked their participants to limit the number of submitted photographs or the researchers themselves have limited the photographs that were discussed to a certain number (e.g. up to five photos) (Ferlatte et al., 2022; Boamah et al., 2022; Diez et al., 2017; Gravina et al., 2020). In this study, we observed that the differences in the number of pictures submitted per participant had no apparent effect on the duration of the discussion or the richness of data generated. On the contrary, the lack of instructions regarding the number of photos enabled the participants to engage freely with the method and encouraged their autonomy and creativity, just as photovoice was originally conceptualised by Wang (Wang et al., 1998).

### Including People From Low-Income Groups in Research

When engaging with people from low-income households, a first challenging step for researchers is identifying potential participants and making them aware of the study or research opportunity. This was possible in this study due to contact building activities previously undertaken by the research team. Partnering with community groups and organisations that worked closely with and offered services to low-income families and single parents was critical to raise awareness of the opportunity for their voices to be heard. Even though our community contacts had to transition to remote work during the COVID-19 pandemic, we were glad to see that this transition did not result in challenges in engaging with them or in any delays in disseminating information about the study to their networks.

Additionally, it is important that researchers create a safe space within which people living on a low income can share their stories, something repeatedly highlighted by researchers who work with ‘vulnerable’ groups (elderly in care homes), or address emotive topics (frontline working during the COVID-19 pandemic or experiencing racism) (Ferlatte et al., 2022; Pickering et al., 2002; Rana et al., 2023; Tanhan & Strack, 2020). This can be achieved by being transparent about the objectives of the research work and about participants’ rights to withdraw; being mindful not to burden participants in terms of time and work; and having procedures in place in order to support them should they become upset, such as a distress protocol. Researchers were aware of the need to check participants were happy to continue at regular intervals and to ask permission to discuss sensitive topics. This approach enables on-going consent, checks comfort levels and can put people at ease. Moreover, it can help build trust and rapport which leads to an open and honest conversation (O’Reilly et al., 2011). Furthermore, our participants were given the option to choose their video options (camera on or off), which further contributed to creating an environment where they felt comfortable and safe to share their experiences.

### Maximising the Use of Photovoice in an Online Environment

Apart from its ability as a participatory data collection method to generate rich qualitative findings, our experience is that photovoice can also be an appropriate data collection method that participants can successfully engage with in an online environment. Remote methods such as this can increase participation from groups, for which in-person methods may be more time consuming or inaccessible (e.g. for stay-at-home parents), and/or research budgets may limit scope for travel to rural areas to collect data there. As researchers, being able to expand recruitment beyond our immediate urban environments may have enabled us to get more diverse views. Some of our reflections on the requirements of the online medium and potential participant burden are the following: all participants owned a smartphone for photo-taking and had experience in taking part in online meetings. Familiarity with online communication platforms may have been due to their age, that is, ≤51 years; according to recent reports in the United Kingdom and Ireland, people below the age of 50 are more likely to be confident in using multiple web platforms (Gibney & McCarthy, 2020; Ofcom, 2022).

The online interviews were conducted on *MS Teams*, which at the time of this study required participants, who wished to use their smartphones for the photovoice interviews, to download the *MS Teams* application in advance of the interview. This did not seem to be a burden to any of the participants who all willingly downloaded the platform or were already familiar with it. However, in the case that participants did not wish to download it, the alternative of a regular phone call had been considered. Furthermore, participants did not require any assistance with technical aspects of the study conduct (taking and sharing photographs or logging onto MS Teams). The research team was however available to connect with them outside of scheduled study meetings to assist with any technical difficulties they may encounter. It is important however to note that employing online study designs, as in this exploration, might be time consuming for and cause fatigue to people with less technological skills, but also exclude from participation people with unstable (or no) internet and electricity connection such as individuals in emergency settings (Oliffe et al., 2023; Paulus & Lester, 2021).

### Additional Ethical and Data Safety Considerations

Even though most of the fundamental ethical issues in online interviewing are the same as in face-to-face contexts (e.g. confidentiality and need for anonymisation of transcripts/questionnaires), conducting online interviews means that additional ethical issues and data protection matters need to be addressed, such as data-sharing protocols and obtaining consent remotely. In this study, participants were sent an electronic file of the consent form and were asked to provide their informed consent on this file and return it through email. This did not seem to be a problem for this sample, and all participants completed the consent form by typing their name, by hand signing it (on an electronic or a printed document) or even by responding to the researcher’s email indicating consent. Relevant literature proposes various ways to obtain remote consent, which were considered in the present study as alternatives. One such alternative is emailing participants with the consent form in the body of the email and request that the participants reply to that message as an expression of consent (Lobe, 2017). Researchers can also utilise online survey tools (e.g. SurveyMonkey or Qualtrics) for the purpose of obtaining consent, whereby the participants are sent a link, unique to them, that leads to a webpage with the consent form which can be then ticked or initialled, as employed by Tanhan and Strack (2020) and by Doyumğaç and colleagues (2021). Ultimately, when considering pathways to obtain consent, it is important to remain flexible and ensure that the methods used are not exclusionary while meeting the ethical and data regulation processes.

In relation to photo sharing options, our experience demonstrates that participants needed different options to enable them to share their photos and stay engaged in the study. WhatsApp is generally considered a secure messaging platform due to its built-in end-to-end encryption, meaning that data exchanged between two users can be accessed only by them (Barbosa & Milan, 2019). Diez and colleagues chose another approach to sharing data by offering digital cameras to their participants, which they retrieved (along with the saved pictures), when data collection was finished (Diez et al., 2017), something that was impossible for the present study due to the COVID-19 restrictions. Experience gained during this study and by Oliffe (2023) highlights the need for flexibility around obtaining consent and data sharing (whilst maintaining ethical standards), as leaving participants with limited options, which they may not be familiar with, can compromise the participatory ethos of the photovoice exercise and even discourage them from participating. Increased (data sharing) regulation, stringent ethical review and the aversion to risk need to be balanced with the practical application of research, including gaining and documenting informed consent and sharing experiences. Researchers have an obligation to develop ethical literacy and sensitivity in the conduct of their research, and ethics committees should have corresponding trust in researchers’ abilities in the field (Miller & Boulton, 2007; Hammersley & Traianou, 2014).

### Limitations and Challenges With Sample Selection

Participants for this research self-identified as living on a tight budget, an inclusion criterion that can be open to interpretation. This term was recommended by the parent advisory panel (as opposed to the initially suggested ‘low income’) to ensure a more open and inviting approach for potential participants. We believe that despite the online environment, parents talked openly about their daily lives and their financial constraints in relation to their food decisions. Indeed, most were on state benefits, confirming that the perspectives captured in this study reflect the experiences of low-income households on the island of Ireland.

Additionally, it should be noted that our study was limited in terms of capturing the perspectives of fathers. This is in line with previous research which highlights that services that facilitate recruitment for parent-focused research are mainly utilised by mothers and are often not accessible or attractive to males (Fletcher & StGeorge, 2010). However, given that family dynamics are complex and that both parents’ perspectives are important (Cabrera et al., 2018), alternative recruitment approaches that are specific to males should be considered (Davison et al., 2017).

## Conclusion

In this methodological article, we provided reflections on utilising the method of online photovoice to better understand how low-income families navigate their food environment. Our experience is that photovoice offers an effective, engaging and flexible tool that can generate rich qualitative data highlighting issues that can form the priorities of healthcare policy, which may be otherwise overlooked by other research methodologies. Practical considerations around ethics and online data collection and sharing should include the target population, their ability to use remote technologies and whether the research topic can be emotive for them, as well as data safety of the available online tools. The involvement of a parent panel was also instrumental at the outset of the study.

## Implications of This Research

The methodological reflections outlined here provide some key considerations which are pertinent to researchers and healthcare practitioners who work with socioeconomically deprived groups and wish to employ photo-elicitation in an online, flexible, safe and non-intrusive way. They also provide a guide for research aiming to understand better food decisions, an area that is extremely salient given the energy crisis and subsequent drop in food affordability following the pandemic, and hold potential to be applied in other research contexts.

## Supplemental Material

Supplemental Material - Using Online Photovoice to Explore Food Decisions of Families on Low Income: Lessons Learnt During the COVID-19 Pandemic Click here for additional data file. Supplemental Material for Using Online Photovoice to Explore Food Decisions of Families on Low Income: Lessons Learnt During the COVID-19 Pandemic by Eleni Spyreli, Elena Vaughan, Michelle C. McKinley, Jayne V. Woodside, Marita Hennessy, and Colette Kelly in Qualitative Health Research

Supplemental Material - Using Online Photovoice to Explore Food Decisions of Families on Low Income: Lessons Learnt During the COVID-19 Pandemic Click here for additional data file. Supplemental Material for Using Online Photovoice to Explore Food Decisions of Families on Low Income: Lessons Learnt During the COVID-19 Pandemic by Eleni Spyreli, Elena Vaughan, Michelle C. McKinley, Jayne V. Woodside, Marita Hennessy, and Colette Kelly in Qualitative Health Research

Supplemental Material - Using Online Photovoice to Explore Food Decisions of Families on Low Income: Lessons Learnt During the COVID-19 Pandemic Click here for additional data file. Supplemental Material for Using Online Photovoice to Explore Food Decisions of Families on Low Income: Lessons Learnt During the COVID-19 Pandemic by Eleni Spyreli, Elena Vaughan, Michelle C. McKinley, Jayne V. Woodside, Marita Hennessy, and Colette Kelly in Qualitative Health Research
