# Supplementary material for: Using Online Photovoice to Explore Food Decisions of Families on Low Income: Lessons Learnt During the COVID-19 Pandemic
Source: Qual Health Res. 2023 Nov 7;34(3):171–82. doi: 10.1177/10497323231208829 (PMC10768336; doi:10.1177/10497323231208829)
Supplement: Supplemental Material - Using Online Photovoice to Explore Food Decisions of Families on Low Income: Lessons Learnt During the COVID-19 Pandemic [file sj-pdf-1-qhr-10.1177_10497323231208829.pdf]

Questions to facilitate photo-elicitation

Key questions for *each* photograph:

- Can you describe your photo?
- What is happening in this photo?
- Why did you take a photo of this?
- Would you take this picture prior to the COVID lockdown?
    - If not, why?
    - If yes, would there be any differences?
