# Supplementary material for: Using Online Photovoice to Explore Food Decisions of Families on Low Income: Lessons Learnt During the COVID-19 Pandemic
Source: Qual Health Res. 2023 Nov 7;34(3):171–82. doi: 10.1177/10497323231208829 (PMC10768336; doi:10.1177/10497323231208829)
Supplement: Supplemental Material - Using Online Photovoice to Explore Food Decisions of Families on Low Income: Lessons Learnt During the COVID-19 Pandemic [file sj-pdf-2-qhr-10.1177_10497323231208829.pdf]

Introducing participants to Photovoice

Taken from the section of Participant Information Sheet entitled "What will happen to me if I take part?"

We will ask you to take part in two interviews two of which will take place over the phone or a safe online communication platform. The first interview will be very short and will introduce you to the type of research we are conducting. You will also be asked a few questions so we can get some more information about you.

You will then be asked to take photographs related to your experience of making decisions about food for your family. Photos will be taken over the period of one week and they can illustrate where you shop, offers on food, marketing, taste, your budget, your receipts and anything else you would like to capture. These photographs will then be shared via email and discussed with our researchers at a second interview.

We are asking you to take photographs of anything that influences what you choose to buy or prepare for your family.

This includes photographs of:

- What you/your family eat
- Things that influenced your family food choice, e.g. offers, marketing, shops
- Where you/your family buy food
- Where you/your family eat, e.g. in café or local restaurant
- Receipts for foods/meals
- Anything else you think is important about food, and your experience of shopping and planning food for your family

You are asked to use your own camera phone to do this. If you're unsure about taking photographs with your phone, please tell us and we can show you how to do so.
