# Supplementary material for: Using Online Photovoice to Explore Food Decisions of Families on Low Income: Lessons Learnt During the COVID-19 Pandemic
Source: Qual Health Res. 2023 Nov 7;34(3):171–82. doi: 10.1177/10497323231208829 (PMC10768336; doi:10.1177/10497323231208829)
Supplement: Supplemental Material - Using Online Photovoice to Explore Food Decisions of Families on Low Income: Lessons Learnt During the COVID-19 Pandemic [file sj-pdf-3-qhr-10.1177_10497323231208829.pdf]

Examples of participants' photos

The following photographs were chosen as exemplary of the various categories of pictures received during the data collection stage of this study. They are accompanied by an informative quote the participants shared when discussing the photo.

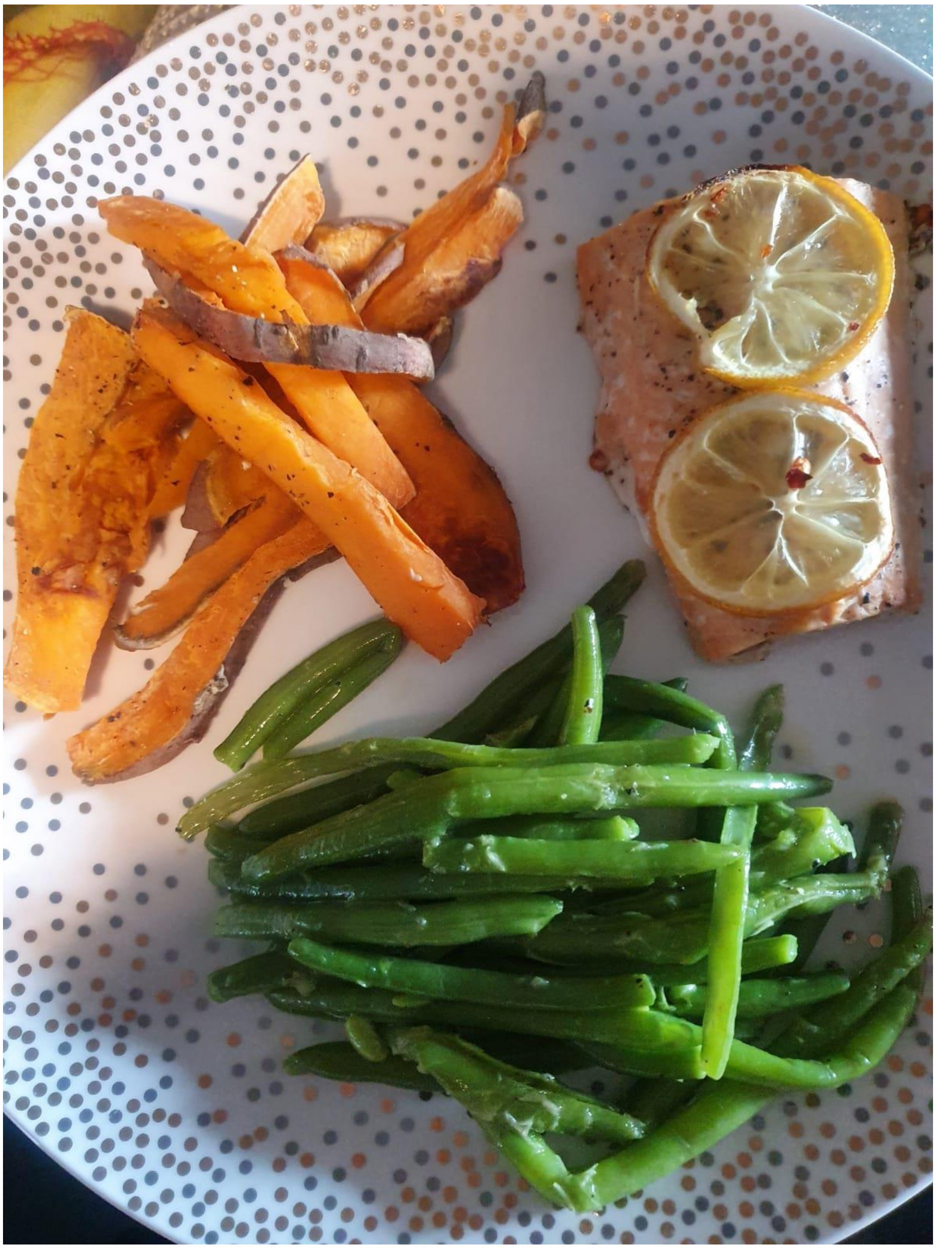

Figure 1. Meals served

And then again my mom and dad had to give it [salmon] to us, cause they had just spare… So we would help each other out too. … cause I'm not working, my dad's not working at the moment either so both households are a wee bit strapped for cash at the minute …
[Female, married]

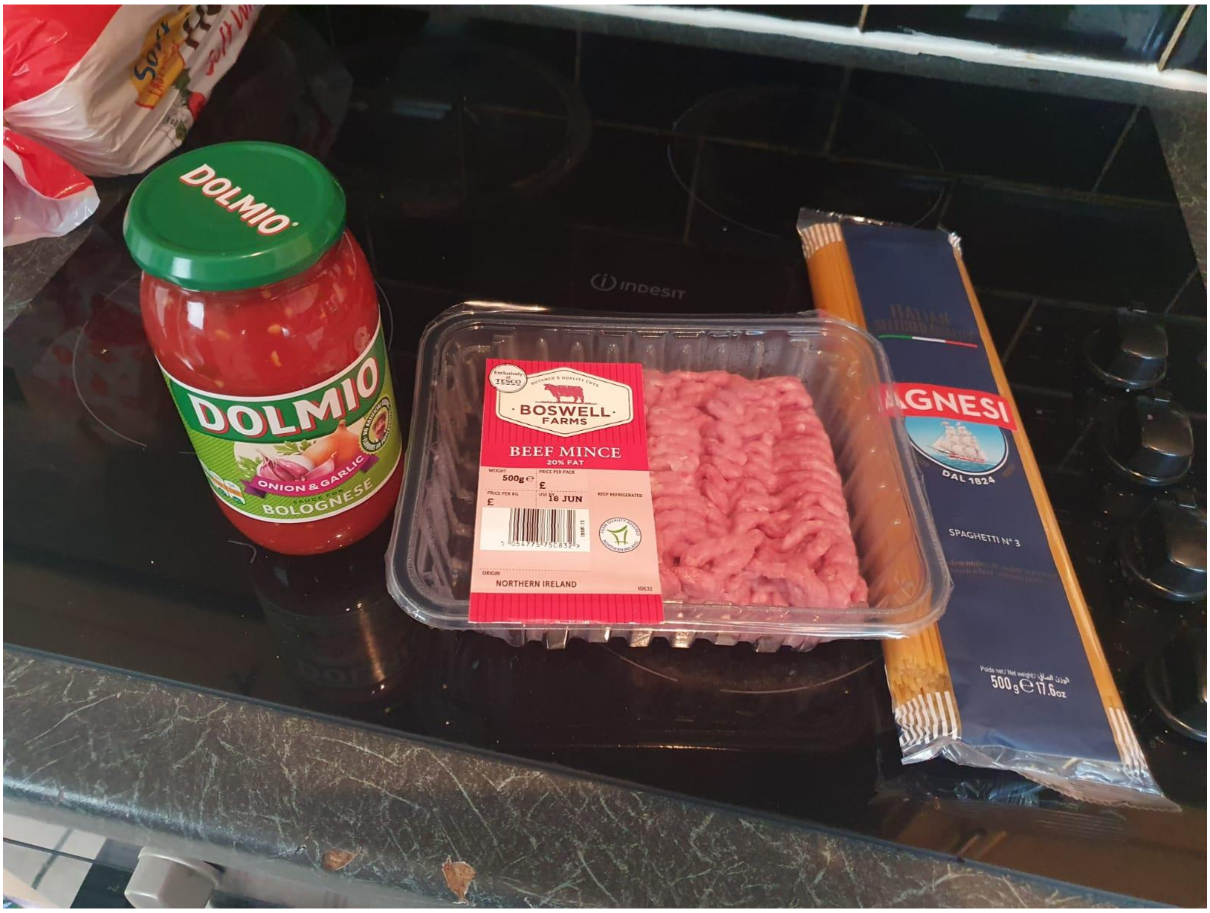

Figure 2. Foods purchased

My cooking skills are pretty limited... So now, you know, for my spaghetti Bolognese, I will use the Dolmio sauce. [Male, single]

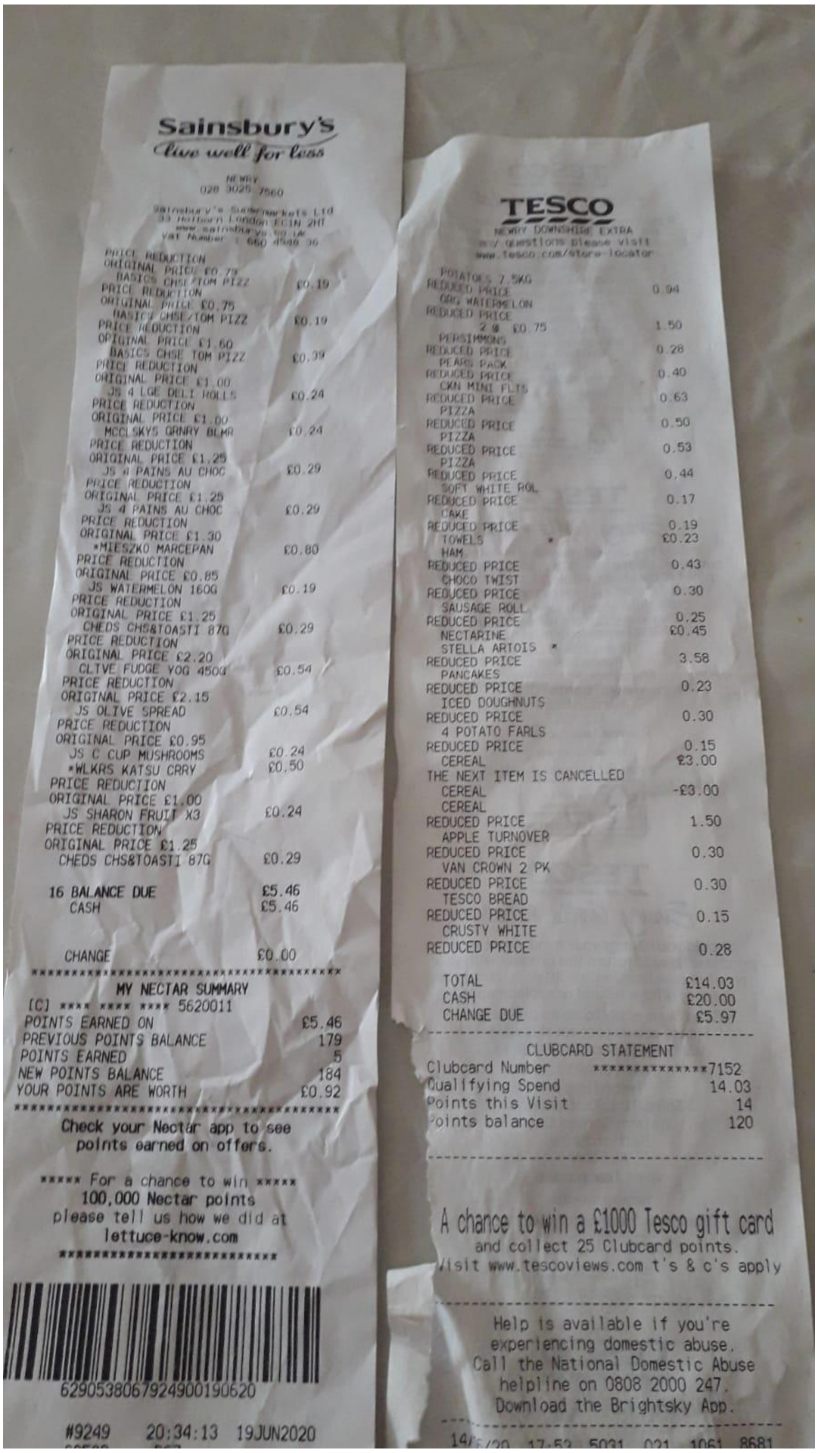

Figure 3. Receipts from food shopping

[I shop from Tesco] Because they have a… Polish stuff as well. Like I love some Polish stuff there and as well as some good stuff, like reduce stuff. [Female, married]

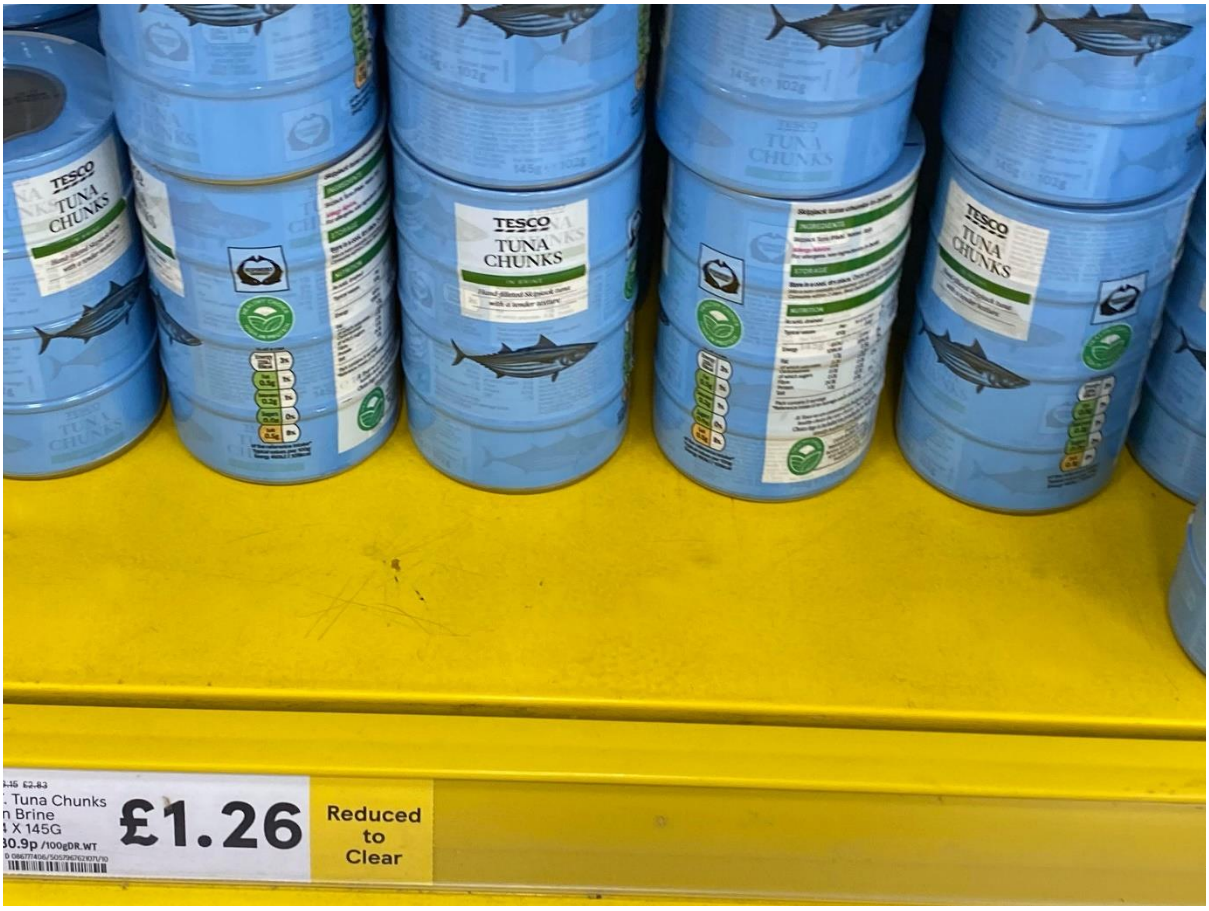

Figure 4. Supermarket promotions

That's a bargain and see if I see stuff like that, I would pick maybe four of them up and keep them 'cause it's not very often you see a bargain like that on tuna. [Female, single]

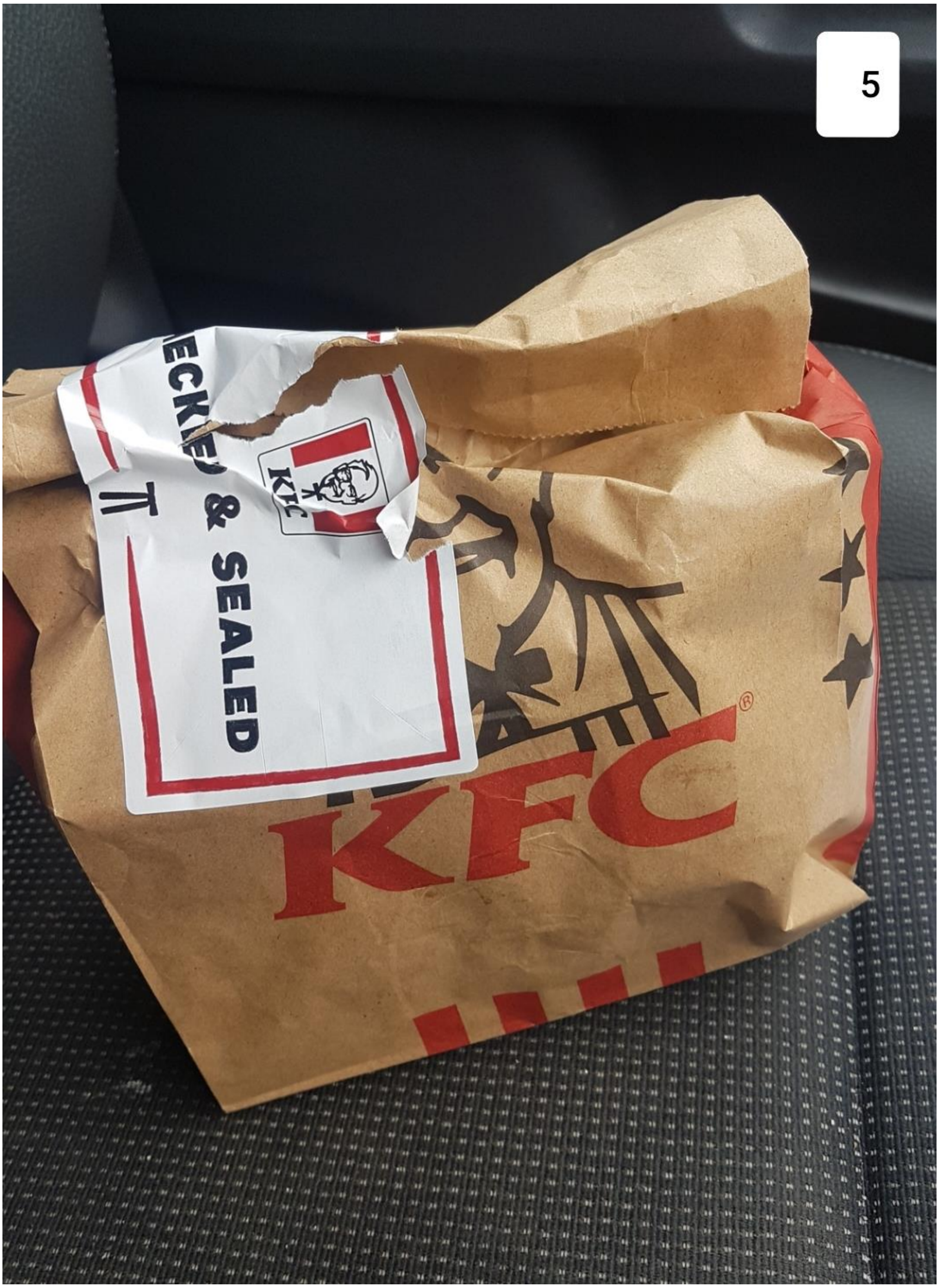

Figure 5. Takeaways

I was coming home to the older boys. So just I know they love KFC and it's only recently reopened as well. So I just went through the drive-through and brought them home some food
[Female, married]

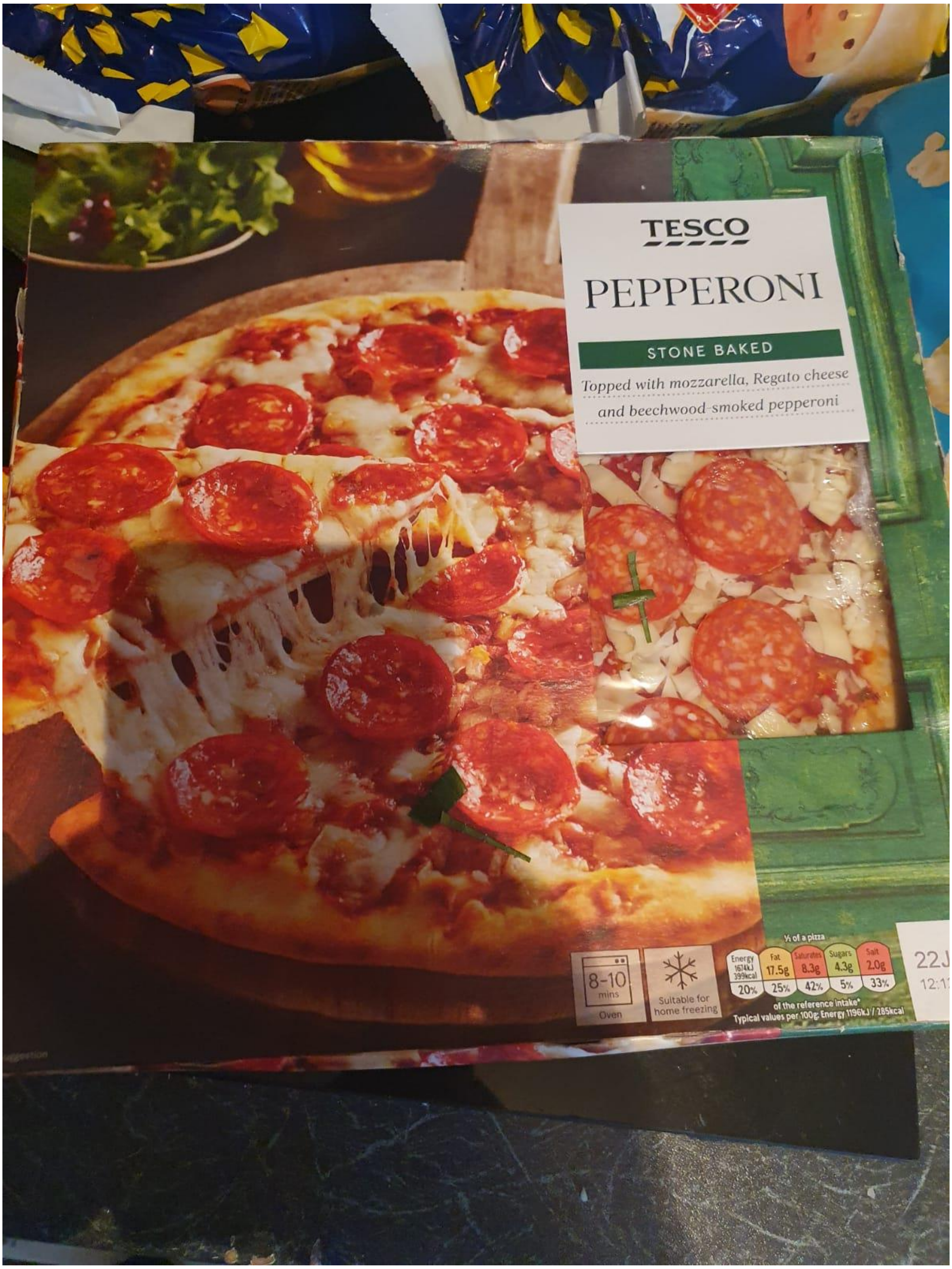

Figure 6. Convenience meals

Pizzas: it's a generally a crowd pleaser in my family. [Male, single]

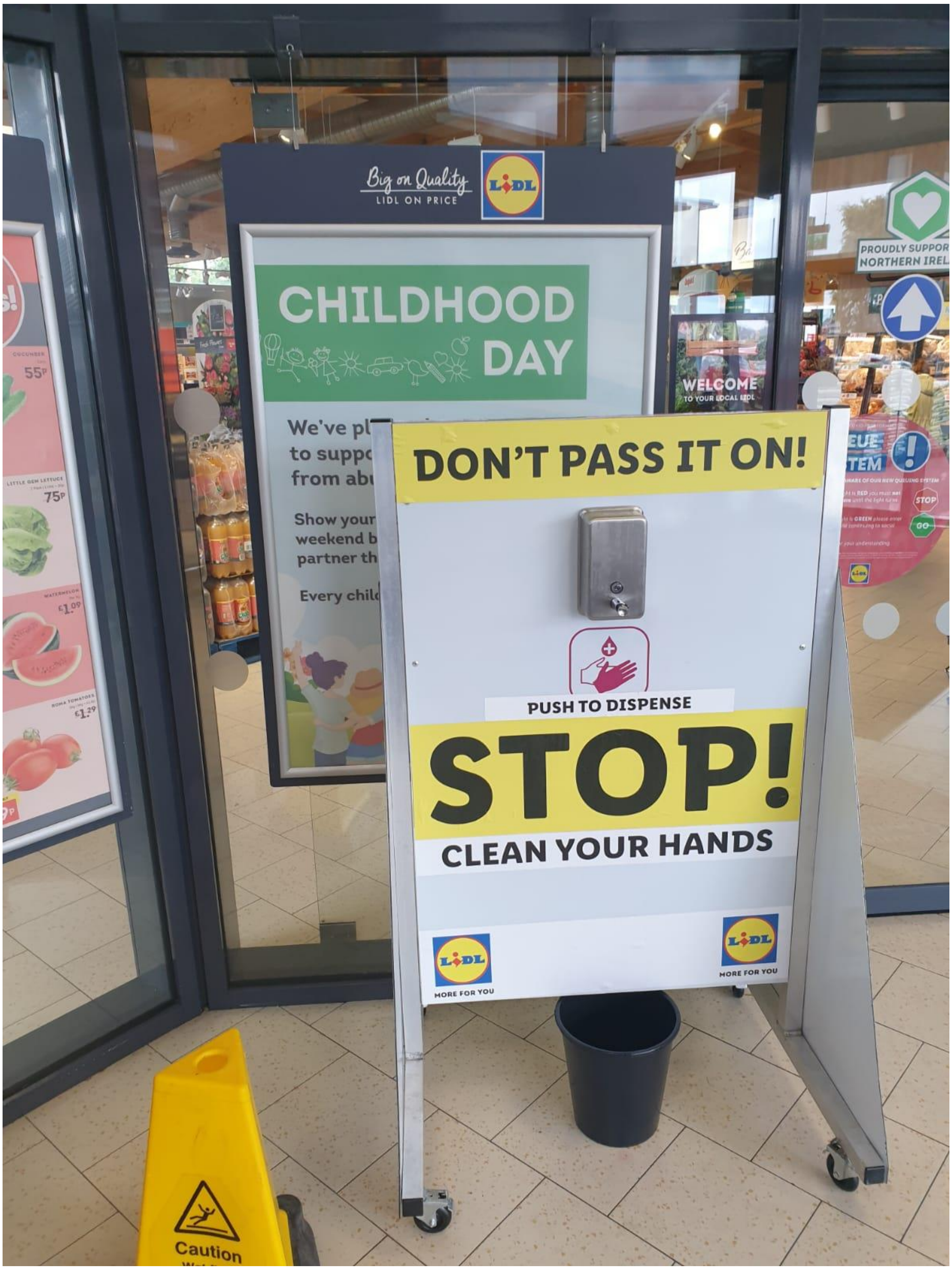

Figure 7. Supermarket COVID-19 adjustments

[They] had all the restrictions in place, you know lines and things and indicators on the floor. So it was really well-prepped. I felt comfortable going in there. [Female, married]
